# Surgical Treatment of Childhood Pulmonary Hydatidosis: An Analysis of 25 Cases

**Published:** 2018-10

**Authors:** Miktat Arif Haberal, Erkan Akar, Ozlem Sengoren Dikis, Mete Kaya

**Affiliations:** 1 Health Sciences University, Bursa Yuksek Ihtisas Training and Research Hospital, Department of Thoracic Surgery, Bursa, Turkey,; 2 Health Sciences University, Bursa Yuksek Ihtisas Training and Research Hospital, Department of Pulmonary Diseases, Bursa, Turkey,; 3 Health Sciences University, Bursa Yuksek Ihtisas Training and Research Hospital, Department of Pediatric Surgery, Bursa, Turkey

**Keywords:** Chest surgery, Childhood, Cystotomy, Capitonnage, Enucleation

## Abstract

**Background:**

Hydatid cyst disease is caused by the parasite *Echinococcus granulosus* and it is an important health problem in the childhood period. In the present study, we aimed to report our experience in 25 surgically managed pediatric hydatid cyst cases under the light of the relevant literature.

**Materials and Methods:**

We retrospectively analyzed 25 patients below 15 years of age who were treated for pulmonary hydatid cyst at our clinic between 2005 and 2016. The patients were analyzed for age, sex, signs and symptoms, diagnostic methods, cyst localization, diameter, number, treatment modalities, mortality, morbidity, and recurrences.

**Results:**

Of the 25 patients included in this clinical study, 16 were male and their mean age was 10.5 (range 5–15) years. The most common presenting symptom was paroxysmal cough which affected 18 patients. The cysts were located in lungs in 23 patients and lungs and liver in 2 patients. Nineteen pulmonary cysts were solitary, and 21 (66%) were in the lower lobe. Thirteen (52%) patients had perforated cysts. Fourteen (56%) patients were operated with cystotomy and capitonnage, 9 (36%) with cystotomy, and 2 (8%) with enucleation. No case of recurrence was observed during an average 12 (range 8–18) months of follow-up.

**Conclusion:**

Surgery is the primary treatment of pediatric pulmonary hydatid cyst disease. Cystotomy and capitonnage is the most commonly used parenchyma sparing technique.

## INTRODUCTION

Hydatid Cyst (HC) disease is caused by the parasite *Echinococcus granulosus*. It is particularly common in societies engaged in agriculture and livestock breeding, and is endemic to many regions around the world. The disease is transmitted via fecal-oral route, with humans being an intermediary host in the disease’s cycle. Whereas the cysts most commonly reside in the liver, pulmonary involvement is more common in children (1,2). Pulmonary cysts are more commonly located in the lower lobe of the right lung (1). As the disease’s clinical signs and symptoms depend on a cyst’s size, they may remain subtle for prolonged periods. The primary symptoms of pulmonary involvement are chest pain, cough, and hemoptysis (1,2).

In the present study, we aimed to report our experience in 25 surgically managed pediatric HC cases under the light of the relevant literature.

## MATERIALS AND METHODS

We retrospectively analyzed 25 patients below 15 years of age who were treated for pulmonary HC at our clinic between 2005 and 2016. Patients’ medical records were accessed through our hospital’s automation system. Demographic properties including age, sex, signs and symptoms were analyzed. The results of complete blood count, biochemistry, posteroanterior chest radiography, thoracic Computerized Tomography (CT), and abdominal ultrasonography were reviewed. Cysts localization was analyzed in terms of surgical technique and postoperative complications. Having a better intestinal absorption, albendazole was administered to 20 patients at a dose 10 *mg/kg* for 4–8 weeks to reduce the recurrence rate and disease infectivity.

### Operative Technique

All patients were operated under general anesthesia at the operating theatre. They were positioned on the operating table according to cysts’ locations (right/left thoracotomy position). Thoracic cavity was entered via posterolateral thoracotomy. In order to prevent cysts’ spread to adjacent tissues, they were wrapped with “1.5% cetrimide-0.15% chloride hexidin” (10% Savlon) soaked gauzes. The walls of unperforated cysts were perforated with a cannula connected to an aspirator’s end, and cyst fluid was aspirated without spilling it over the surrounding tissues. Then, cystotomy was performed and membranes in the cavity were totally excised. As for perforated cysts, the cystotomy procedure was directly applied. No scolocidal agent was injected into cyst cavity due to the risk of tracheobronchial irritation. Distilled water was poured into cyst cavity to check for bronchial leaks. The bronchial ostia with leaks were sealed with absorbable surgical sutures (poliglactyn, 2/0, 3/0), and then cyst cavity was quilted. Air leaks and hemostasis were checked, a single drain was placed into thorax, and the layers were closed on anatomic plane. The patients were extubated on the operating table and then sent to regular ward.

### Statistical analysis;

The study data were analyzed using SPSS for Windows (IBM 21.0) software package. Student t test was used for comparisons of age and sex. Fisher’s exact test was used to compare cyst localization by patient sex. The comparison of complications between the surgical methods was done with Chi-Square test. A P value of less than 0.05 was considered statistically significant for all statistical comparisons.

## RESULTS

**Figure 1.**
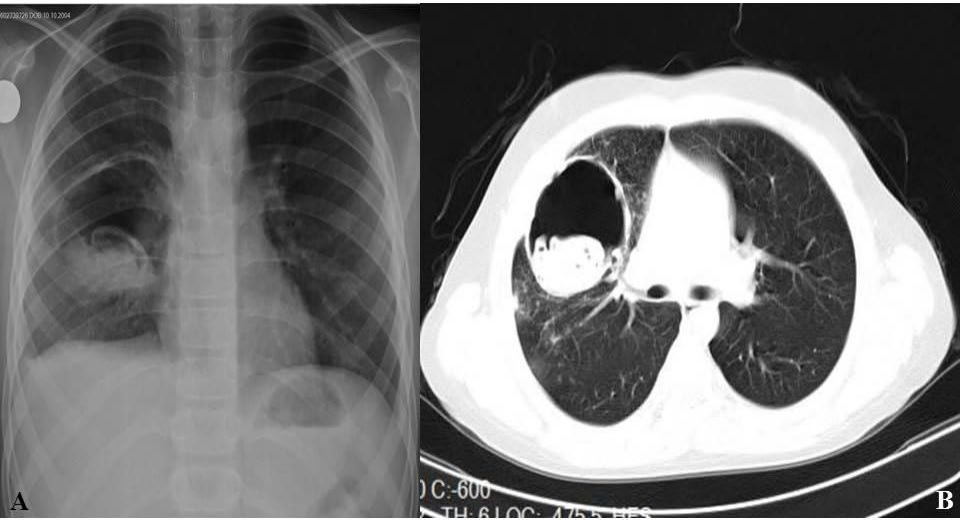
A) The postero-anterior chest radiogram shows a perforated cystic with air fluid levels in the right lung. B) The thoracic computerized tomography shows a cystic

Sixteen patients were male and 9 were female. The mean age was 10.5 (range 5–15) years. The most common symptom in patients with pulmonary HC was paroxysmal cough (Table 1) followed by watery expectoration from mouth. Cysts were located in lungs in 23 patients and both lungs and liver in 2 patients. The diagnosis of the condition was made by PA chest radiograms and thoracic CTs in addition to clinical signs and symptoms in all patients (Figure 1). Pulmonary cysts were solitary in 19 cases, and 21 (50%) cysts were located in the lower lobe. The cysts were ruptured in 13 (52%) of cases, with no allergic or anaphylactic reactions having occurred in patients with ruptured cysts. The pulmonary localizations of hydatid cysts were summarized on table 2.

**Table 1.** Patient’s symptoms

**Symptoms**	**N**	**%**
**Cough**	18	35
**Expectoration**	12	23
**Chest Pain**	9	18
**Hemoptysis**	4	8
**Shortness of breath**	8	16
**Total**	51	100

**Table 2.** Pulmonary localizations of the hydatid cysts

**Localization**	**N**	**%**
**Right lower lobe**	12	38
**Right upper lobe**	5	16
**Right middle lobe**	4	12
**Left lower lobe**	9	28
**Left upper lobe**	2	6
**Total**	32	100

All patients were operated with posterolateral thoracotomy. Fourteen (56%) patients were operated with cystotomy and capitonnage, 9 (36%) with cystotomy, and 2 (8%) enucleation. No patient underwent parenchymal resection like lobectomy. In patients with bilateral pulmonary involvement the cyst in the contralateral lung was also surgically treated 4–6 weeks later. During postoperative follow-up, 2 patients developed prolonged air leak, 1 developed atelectasis, and 1 developed wound infection. The mean duration of hospital stay was 7 (range 4–10) days. No case of recurrence was observed during a follow-up period of 12 (range 8–18) months. There were no significant differences between age, sex, cyst’s pulmonary localization and operative techniques.

## DISCUSSION

HC disease is an important parasitic infection common to all societies where agriculture and livestock breeding is prevalent and environmental health measures and preventive healthcare services are inadequate. The disease prevalence shows significant variation between countries and regions. Various studies have put the disease prevalence between 1 and 500 per 100.000 population. The disease incidence in our country is about 20 per 100,000 and its prevalence is about 50 per 100,000 (3,4).

Whereas hydatidosis is more common among women than men in adults, male children are more commonly are reportedly affected more commonly than their female counterparts (5). Most of our patients were male, with a male/female ratio of 1.8.

Although HC most commonly involves liver, pulmonary involvement is more common in children (1,2, 6). The majority of cysts are asymptomatic. Symptoms emerge depending on cyst enlargement and involved organ (7). Due to the delay in the diagnosis, the cysts were detected as larger sized cysts (8). Five of our 12 patients had a cyst size equal to or greater than 5 *cm*. Pulmonary involvement of HC most commonly causes chest pain, cough, and hemoptysis (4,9). Our patients’ symptoms were consistent with previous reports.

Conventional chest radiograms are the most valuable diagnostic modality for diagnosing pulmonary HC. Serological tests combined with chest radiogram aid clinicians for reaching a definitive diagnosis. However, radiological imaging tests are more reliable than serological tests (9). Thoracic CT is valuable for detecting cysts that are invisible in PA chest radiograms or determining the density of intact cysts (9). We also managed to diagnose the condition with the help of these two modalities.

In chest radiogram, ovoid or spherical opacities are diagnostic of non-complicated pulmonary cysts while air-fluid level or more specific signs such as lotus or meniscus sign are diagnostic for complicated cysts (1). In a 196-case series were diagnosed the disease with chest radiograms in 132 cases, tomographic examinations in 40 cases, and ultrasonography in 24 cases (6).

It was reported that lung involvement is usually solitary but may be multiple in 14–30% of cases, with the disease favoring the lower lobes (10,11). In a series comprising 1055 cases it was reported that 60% of cases were right-sided, 38% were left-sided, 2% were bilateral, and 75 patients had multiple pulmonary cysts (1). In another study it was reported that cysts were left-sided in 48.9% of cases, right-sided in 40.3%, and bilateral in 10.8% (6). Our cases were solitary in 21 patients, bilateral in 2 patients, and hepatic and pulmonary combined in 2 patients.

The main treatment for lung cyst is surgery (12). The surgeon’s goal is to remove the cyst with the membrane, to eradicate the parasite without forming a contaminant, and to close the remaining cavity (12). The choice of surgical technique depends on the condition of the tissue surrounding the cyst during surgery. Commonly accepted procedures in surgery are parenchymal protective procedures (13). While all alive parasitic material is removed, especially in children, the intact lung tissue should be preserved to a maximum extent. The most common surgical method is cystotomy and capitonnage (13). We also used it for 14 of our patients.

In bilateral HC, one or two stage surgical approach should be considered. Primarily, the surgery should be performed on the area with the larger cyst or containing a larger number of cysts in the cases of the uncomplicated cysts for which two stage approaches is preferred (14). The cysts were bilateral in two of our cases. There were cysts in the both areas of a case, whereas there was one cyst on the left; two cysts on the right in the other case. Thus, we planned our priority according to the area with larger cyst and containing a larger number of cysts as indicated in the literature.

The most common early complications of isolated HC disease are atelectasis, prolonged air leak, pneumonia, and empyema, and the late complication is recurrent HC disease (11,13). Two of our patients developed prolonged air leak, one developed atelectasis, and another one developed wound infection.

As an alternative to surgical treatment, benzimidazole derivatives, which is used for the medical therapy of HC disease, are recommended for four days before and 1–3 months after surgery to minimize the spread of cyst content in an attempt to prevent anaphylactic reactions and secondary recurrences (15). We used albendazole at a dose of 10 *mg/kg* in 20 cases for 4–8 weeks postoperatively in an attempt to prevent disease recurrence and spread.

HC disease usually portends a favorable prognosis. Depending on a cyst’s localization and surgeon’s experience, the recurrence rates vary between 2 and 25%. Depending on the same factors, operative mortality ranges between 0.5 and 4% (13). No case of recurrence or death occurred in our study population.

Limitations of our study include retrospective evaluation of events and failure to reach all data related to events.

## CONCLUSION

HC is common in our country, and pulmonary involvement is more prevalent among children. Pulmonary HC disease should be frequently considered in the differential diagnosis of patients with nonspecific symptoms such as chest pain, hemoptysis, and fever, and radiological signs of the disease. The disease is surgically treated, with parenchyma sparing techniques being preferred in the pediatric age group.

### Scientific Responsibility Statement

The authors declare that they are responsible for the article’s scientific content including study design, data collection, analysis and interpretation, writing, some of the main line, or all of the preparation and scientific review of the contents and approval of the final version of the article.

### Animal and human rights statement

All procedures performed in this study were in accordance with the ethical standards of the institutional and/or national research committee and with the Helsinki declaration and its later amendments or comparable ethical standards.

## References

[1] Arslan S Özşahin SL Doğan T Akkurt İ Multiple complicated lung hydatid cyst associated with interatrial septal hydatid cyst. Ege Journal of Medicine 2009;48:135–7.

[2] Oudni-M’rad M M’rad S Babba H Molecular and epidemiology data on cystic echinococcosis in Tunisia. InCurrent topics in echinococcosis 2015 InTech.

[3] Mahmodlou R Sepehrvand N Nasiri M Saucerization: a modified uncapitonnage method of surgery for pulmonary hydatidosis. World J Surg 2013;37(9):2129–33. 23657752 10.1007/s00268-013-2093-7

[4] Aydogdu B Sander S Demirali O Guvenc U Besik C Kuzdan C Treatment of spontaneous rupture of lung hydatid cysts into a bronchus in children. J Pediatr Surg 2015;50(9):1481–3. 25783398 10.1016/j.jpedsurg.2015.01.010

[5] Irfan KJ Shivanna S Sudhir U Kempegowdaet P Primary Pulmonary Echinococcosis: A Case Report. West London Medical Journal 2014;6:29–32.

[6] Çelik T Akçora B Tutanç M Yetim TD Karazincir S Akın MM Ruptured pulmonary hydatid cyst: a case report. Turkiye Parazitol Derg 2012;36(1):45–7. 22450922 10.5152/tpd.2012.11

[7] Döleş K Eren Ş Döleş FT Kukul MG Korkmaz İ Coşkun A A case of cyst hydatid have a family history with pulmonary, hepatic and splenic involvement. Cumhuriyet Med J 2009;31:288–92.

[8] Katrancıoğlu Ö Şahin E Karadayı Ş Kaptanoğlu M Diagnosis and treatment approaches of the lung hydatid cysts in childhood. Current Thoracic Surgery 2017;2(1).

[9] Gayretli Aydın ZG Yalçınkaya R Aydın Teke T Bayhan Gİ Öz FN Metin Timur Ö Coexistence of Pulmonary Hydatid Cyst and Mycoplasma pneumoniae Pnömonia in a Child. Turkiye Parazitol Derg 2015;39(2):159–63. 26081892 10.5152/tpd.2015.3609

[10] Koca T Dereci S Gençer A Duman L Aktaş AR Akçam M Cystic Eechinococcosis in Childhood: Five-Years of Experience From a Single-Center. Turkiye Parazitol Derg 2016;40(1):26–31. 27222332 10.5152/tpd.2016.4381

[11] Usluer O Ceylan KC Kaya S Sevinc S Gursoy S Surgical management of pulmonary hydatid cysts: is size an important prognostic indicator? Tex Heart Inst J 2010;37(4):429–34. 20844615 PMC2929855

[12] Özdemir A Bozdemir ŞE Akbiyik D Daar G Korkut S Korkmaz L Anaphylaxis due to ruptured pulmonary hydatid cyst in a 13-year-old boy. Asia Pac Allergy 2015;5(2):128–31. 25938078 10.5415/apallergy.2015.5.2.128 PMC4415179

[13] Akar E Çakmak M Surgical therapy in patients with pulmonary hydatidosis. İzmir Göğüs Hastanesi Dergisi 2014;28(1):9–13.

[14] Eroğlu A Kürkçüoğlu C Karaoğlanoğlu N Bilateral multiple pulmonary hydatid cysts. Eur J Cardiothorac Surg 2003;23(6):1053. 12829089 10.1016/s1010-7940(03)00150-7

[15] Köroğlu M Erol B Gürses C Türkbey B Baş CY Alparslan AŞ Hepatic cystic echinococcosis: percutaneous treatment as an outpatient procedure. Asian Pac J Trop Med 2014;7(3):212–5. 24507642 10.1016/S1995-7645(14)60023-7

